# Elevated Biomarkers of Inflammation and Coagulation in Patients with HIV Are Associated with Higher Framingham and VACS Risk Index Scores

**DOI:** 10.1371/journal.pone.0144312

**Published:** 2015-12-07

**Authors:** Sarah Mooney, Russell Tracy, Turner Osler, Christopher Grace

**Affiliations:** 1 Department of Medicine, Infectious Disease Unit, University of Vermont College of Medicine, Burlington, VT, United States of America; 2 Department of Pathology and Biochemistry, University of Vermont College of Medicine, Burlington, VT, United States of America; 3 Department of Surgery, University of Vermont College of Medicine, Burlington, VT, United States of America; University of Pittsburgh Center for Vaccine Research, UNITED STATES

## Abstract

**Background:**

Biomarkers of inflammation and altered coagulation are of increasing interest as predictors of chronic disease and mortality in HIV patients, as well as the use of risk stratification scores such as the Framingham index and the Veterans Aging Cohort Study (VACS) score.

**Methods:**

Demographic and laboratory data for 252 HIV patients were assessed for their relationship with 5 biomarkers: hsCRP, D-dimer, Cystatin C, IL-6 and TNF-alpha. Analysis of variance was used to model the association between the number of elevated biomarkers patients had and their Framingham 10 year cardiovascular risk and VACS scores.

**Results:**

87% of patients were male and 75.7% were virally suppressed (HIV RNA <48 copies/ml). The median and interquartile ranges for each biomarker were: hsCRP 1.65 ug/mL (0.73, 3.89), D-dimer 0.17 ug/mL (0.09, 0.31), Cystatin C 0.87 mg/L (0.78, 1.01), IL-6 2.13 pg/mL (1.3, 3.59), TNF-alpha 4.65 pg/mL (3.5, 5.97). 62.6% of patients had more than one biomarker >75^th^ percentile, while 18.6% had three or more elevated biomarkers. Increased age, cigarette smoking, CD4 counts of <200 cells/mm^3^, Framingham scores and VACS scores were most strongly associated with elevations in biomarkers. When biomarkers were used to predict the Framingham and VACS scores, those with a higher number of elevated biomarkers had higher mean VACS scores, with a similar but less robust finding for Framingham scores.

**Conclusions:**

Despite viral suppression and immunological stability, biomarkers of inflammation and coagulation remain elevated in a significant number of patients with HIV and are associated with higher scores on risk stratification indices.

## Introduction

By 2015, it is projected that more than half of adults living with HIV in the US will be 50 years of age or older [1]. Effective antiretroviral therapy has significantly increased long term survival among adults with HIV infection and life expectancy now approaches that of the general population [2]. Despite this success however, patients with HIV remain at increased risk of non-AIDS related co-morbidities such as cardiovascular disease, liver disease, fractures, cancer, and frailty [3–7]. Multiple co-morbidities are not only more common in patients with HIV infection; they also seem to occur at an earlier age than in the general population [8].

In patients with HIV infection, there is increasing evidence that a persistent inflammatory state, even in those patients with suppressed serum virus levels, is responsible for the early development of these co-morbidities. This persistent inflammation is multifactorial, but thought to be in part due to immune dysregulation, coagulopathy and occult viral replication [9]. Studies have shown that patients with HIV have higher levels of inflammatory and coagulation biomarkers such as high-sensitivity C-reactive protein (hsCRP), D-dimer and interleukin-6 (IL-6), and are associated with poorer outcomes and all-cause mortality [10–13]. It is not known if these elevated levels are directly responsible for disease states or are surrogate markers of an underlying pro-inflammatory state which in turn drives disease. HsCRP, D-dimer and IL-6 have been previously shown to be linked with mortality in HIV infection [10]. Elevated D-dimer levels have been associated with an increased risk of cardiovascular disease and hsCRP and IL-6 have been associated with cardiovascular disease and opportunistic infection [11]. Cystatin C is a marker of renal dysfunction that has previously been found to be elevated in patients with HIV compared to the general population [12]. TNF-alpha (TNF-a) has been recently associated with an increased risk of non-AIDS defining conditions [14].

There is increasing interest in the use of these biomarkers as screening tools to identify those at highest risk of non-HIV related co-morbidities, possibly in combination with other routinely monitored clinical variables such as hemoglobin and liver function tests [15]. The role of risk stratification scoring systems, such as the Framingham cardiovascular risk score and the Veterans Aging Cohort Study (VACS) risk score, and their association with markers of inflammation, is also an area of study. The VACS score has been shown to correlate with IL-6, D-dimer and sCD14 levels [15]. Previous studies have shown that higher levels of soluble markers of endothelial function such as plasminogen activator inhibitor type 1 (PAI-1) in HIV-infected patients correlate with higher Framingham risk scores [16]. The VACS score has been validated in several large cohorts of HIV-infected patients [17,18]. The accuracy of the Framingham score in estimating cardiovascular risk in HIV-infected patients is still uncertain [19].

If biomarkers are to be of clinical use in screening HIV populations for disease risk or to monitor therapeutic response, it would be helpful to first understand the range of baseline abnormalities that may be seen in a typical modern HIV clinic and how these correlate with patient characteristics and standard currently available screening tools. Using data from four HIV clinics throughout the state of Vermont, we characterize five major biomarkers of inflammation and coagulation and explore their association with patient characteristics including demographics, HIV progression, components of the metabolic syndrome, viral co-infections, and two integrated risk indices (the Framingham risk score and the VACS Index).

## Methods

### Ethics Statement

The study was approved by the Institutional Review Board of the University of Vermont, IRB number CHRMS#M12-034. No external funding was used for this study.

### Patients

This was a cross sectional study of HIV patients in care in the Comprehensive Care Clinics in Vermont, four statewide clinics serving about 400 patients. All clinic patients older than 18 years of age who signed informed consent were enrolled between October 2011 and January 2012. Data was collected on 34 patient variables or laboratory results: age, gender, race, HIV risk, years in HIV care, years on ART, first CD4 count in clinic, last CD4 count in clinic, last viral load in clinic, AIDS classification (per CDC criteria), systolic blood pressure, diastolic blood pressure, BMI, waist circumference, current smoker, history of smoking, diabetes mellitus, cardiovascular disease, use of antihypertensive medications, use of statin therapy, use of aspirin therapy, CMV sero-status, HCV sero-status, total cholesterol, LDL, HDL, triglycerides, glucose, hemoglobin, platelets, creatinine, aspartate aminotransferase (AST) and alanine aminotransferase (ALT). Patients were considered to be virally suppressed if the last HIV viral load was <48 copies/mL.

### Biomarker Assays

Two EDTA tubes were drawn per patient, centrifuged to separate plasma (4cc per patient) and stored at -70C until transported for analysis. Five biomarkers were assayed in the Laboratory for Clinical Biochemistry Research at the University of Vermont. IL-6 was measured by ELISA with an assay range of 0.15–2500 pg/mL. HsCRP and Cystatin C were measured using a particle enhanced immunonepholometric assay (BNII nephelometer) with assay ranges of 0.16–1100 ug/mL and 0.046–7.25 mg/L respectively. TNF-a was measured using the Human Serum Adipokine Panel B LINCOplex Kit with an assay range of 0.13–10,000 pg/mL. D-dimer levels were measured with immunoturbidometric methods on the Sta-R analyzer with an assay range of 0.02–20 ug/mL. For analysis, we defined a biomarker as elevated if above the 75^th^ percentile, similar to previous studies of inflammatory biomarkers in HIV-infected populations. High sensitivity CRP cutoff values of <1, 1–3, and >3 ug/mL were used to stratify cardiovascular risk into low, intermediate or high risk categories, correlating with a 10 year cardiovascular disease risk of <10%, 10–20% and >20% respectively. Defined clinical threshold values were not available for IL-6, Cystatin C or TNF-alpha.

### Risk Indices

The Framingham 10 year cardiovascular risk score was calculated using age, gender, total cholesterol, HDL cholesterol, systolic blood pressure, smoking status and use of antihypertensive medication, using a standard on-line calculator available from the National Institute of Health [20]. As this calculator is intended for use in adults who do not have diabetes or pre-existing cardiovascular disease, patients with these conditions were not included in this analysis. The VACS index was calculated using age, CD4 count, HIV-1 RNA, hemoglobin, AST, ALT, platelets, creatinine and HCV seropositivity, using the scoring system developed by the VACS Project Team [18].

### Statistical Analysis

The biomarker measurements were log transformed to obtain normally distributed data, similar to previous biomarker studies [13,21]. Univariate assessment of patient variables as predictors of elevated biomarker levels was performed using Pearson correlation for continuous variables and binary logistic regression for dichotomous variables. Statistical significance was set at an alpha < 0.05. The VASC and Framingham scores were log transformed. The effect of increasing number of elevated biomarkers (defined as those patients with values above the 75^th^ percentile) per person on the VACS and Framingham cardiovascular score was assessed by one way ANOVA.

## Results

Between October 5, 2011 and December 30, 2011, 252 patients were consecutively enrolled. Of the 401 active patients in the clinic during the time period of the study, 149 did not participate; 131 were not seen in clinic during that time, 8 had moved away, 6 refused, 2 were ineligible and blood could not be drawn from 2. Patient variables are shown in Table 1. 87.7% of participants were male. More than three quarters of patients were virally suppressed to <48 copies/mL. The majority of patients (55.9%) had current CD4 counts of >500 cells/mm^3^. 49% of patients were classified as having AIDS using CDC criteria though this included patients who had subsequently undergone immune reconstitution. 19 patients (7.5%) had a CD4 count of <200 cells/mm^3^ at the time of the study. Characteristics of patients in the study group (n = 252) compared with those clinic patients not enrolled (n = 149) were similar, but the study group had fewer women (12.3% vs. 21.6%, p = 0.032) and had greater ART use (89% vs. 82%, p = 0.01).

**Table 1:** Patient characteristics. N = 252.

**Demographics**	
Age, mean (SD)	48 (10.2)
Male (%)	221 (87.7)
Caucasian (%)	208 (82.5)
MSM (%)	161 (63.9)
**HIV care**	
Years in care, mean (SD)	8.5 (6.4)
Years on ART, mean (SD)	7.6 (6.2)
On ART at last visit (%)	225 (89.3)
Initial CD4 count, mean (SD)	448 (323)
<200 (%)	54 (21.4)
200–500 (%)	106 (42.1)
>500 (%)	92 (36.5)
Current CD4 count, mean (SD)	573 (315)
<200 (%)	19 (7.5)
200–500 (%)	92 (36.5)
>500 (%)	141 (55.9)
HIV RNA at last clinic visit, copies/mL	
<400 (%)	224 (88.9)
<48 (%)	193 (76.8)
AIDS diagnosis (%)	124 (49.2)
**Cardiovascular risks**	
BP, mean	121/75
Hypertension (>140/90) (%)	39 (15.5)
On antihypertensive therapy (%)	72 (28.6)
Diabetes (%)	13 (5.2)
Cardiovascular disease (%)	13 (5.2)
History of smoking (%)	128 (50.7)
Currently smoking (%)	82 (32.5)
BMI, kg/m2, mean (SD)	26.5 (5.5)
25–30 (%)	88 (34.9)
30–40 (%)	43 (17.1)
>40 (%)	10 (4)
Waist circumference (inches)	
>40 (men) (%)	64 (25.4)
>35 (women) (%)	16 (6.3)
On statin therapy (%)	62 (24.6)
On aspirin (%)	39 (15.5)
**Co-infections**	
HCV seropositive (%)	30 (11.9)
CMV seropositive (%)	226 (89.7)
**Laboratories**	
Glucose >200 (%)	4 (1.2)
Cholesterol >200 (%)	107 (42.5)
HDL <40 (%)	91 (36.1)
LDL >130 (%)	63 (25)
Triglycerides >150 (%)	124 (49.2)
Hb <10 (%)	3 (1.2)
ALT >72 (%)	18 (28.6)
AST >46 (%)	33 (13.1)
Cr >1.2 (%)	41 (16.3)
Plt <150 (%)	37 (14.7)
Fib-4 >3.25 (%)	11 (4.4)
**Risk stratification scores**	
Framingham 10 year CV risk	
>10 (%)	61 (24.2)
>20 (%)	8 (3.2)
VACS score, median (IQR)	12 (6–28)

Abbreviations: SD, standard deviation; BP, blood pressure; HCV, hepatitis C virus; CMV, cytomegalovirus; HDL, high density lipoprotein; LDL, low density lipoprotein; Hb, hemoglobin; ALT, alanine aminotransferase; AST, aspartate aminotransferase; Cr, creatinine; Plt, platelets; Fib-4, Fibrosis-4 score; VACS, Veterans Aging Cohort Study score; IQR, interquartile range.

The median and interquartile ranges for each biomarker were: hsCRP 1.65 ug/mL (0.73, 3.89), D-dimer 0.17 ug/mL (0.09, 0.31), Cystatin C 0.87 mg/L (0.78, 1.01), IL-6 2.13 pg/mL (1.3, 3.59), TNF-alpha 4.65 pg/mL (3.5, 5.97). 35.1% had a hsCRP of <1ug/mL (low risk), 32% had a hsCRP of 1-3ug/mL (intermediate risk), and 27.9% had a hsCRP of 3-10ug/mL (high risk). Twelve (5%) study patients had a hsCRP of >10ug/mL. Forty percent of the study population had a measured D-dimer of >230 ug/mL, which is greater than the diagnostic cutoff for acute thromboembolic disease. A VACS score was calculated for 247 patients. A Framingham score was calculated for 219 patients; 26 were excluded due to pre-existing cardiovascular disease or diabetes and there was insufficient data for an additional 7 patients.

Of 252 study patients, 158 (62.6%) had at least one biomarker in the >75^th^ percentile, 87 (34.5%) had 2 or more, 47 (18.6%) had 3 or more, 18 (7.1%) had 4 or more, and 8 (3.1%) had all 5 biomarkers in the >75^th^ percentile. Of the 12 patients with a hsCRP of >10ug/mL, only 3 had elevations in all 5 biomarkers. Biomarker elevations were associated with a variety of different patient variables [Tables 2 and 3]. On univariate analysis of continuous variables using a p-value cutoff of 0.05, variables with the strongest association with biomarker levels were: age, initial CD4 count, triglyceride levels, Framingham score, and VACS score. The VACS score was strongly associated with four out of the five biomarker levels with p<0.0001. Using a more stringent p-value cutoff of 0.01, these variables remained strongly associated with biomarker levels. On univariate analysis of dichotomous variables, variables with the strongest association with biomarker levels were: AIDS, current CD4 <200 cells/m^3^, smoking (past or present), and co-infection with HCV. HIV RNA did not correlate with elevated biomarker levels, either as a continuous variable or when stratified using a cutoff of 48 or 400 copies/mL. Other HIV-related variables such as years spent in care and years on antiretroviral therapy were not associated with elevated biomarkers. Components of the metabolic syndrome (central obesity, hypertension, hyperglycemia, elevated triglycerides, low HDL) were not particularly associated with elevations in biomarkers, with the exception of elevated triglyceride levels.

**Table 2:** Correlation of biomarkers (log transformed) with continuous variables. P<0.05.

Variable	CRP	Cystatin	D-dimer	IL-6	TNF-a
	P value	P value	P value	P value	P value
Systolic BP	0.06	0.44	**0.02**	**0.006**	0.91
Diastolic BP	0.27	0.83	0.48	0.18	0.73
BMI	0.12	**0.02**	0.82	0.67	0.08
Waist circumference	0.03	0.95	**0.04**	0.10	0.59
Years in care	0.16	0.07	0.10	**0.02**	0.24
Age	0.59	**<0.0001**	**0.0001**	**0.002**	**0.03**
Years on ART	0.07	0.08	0.06	**0.002**	0.11
Initial CD4	0.08	**0.003**	0.60	**0.01**	**0.01**
Δ CD4	0.09	0.80	0.91	0.91	0.76
HIV RNA	0.89	0.33	0.40	**0.02**	0.06
Glucose	0.13	0.89	0.21	0.51	0.41
Cholesterol	**0.0002**	0.16	0.19	0.74	0.27
HDL	0.88	0.18	0.32	0.14	0.12
LDL	**0.04**	0.36	0.64	0.47	0.21
Triglycerides	**0.001**	**0.007**	**0.02**	**0.003**	**0.005**
Hemoglobin	0.15	0.22	**0.01**	0.07	0.62
AST	**0.04**	0.37	0.41	0.50	0.42
ALT	0.21	**0.02**	0.14	0.12	**0.03**
Creatinine	0.66	**<0.0001**	0.93	0.10	**0.002**
Platelets	0.22	0.92	0.86	0.91	0.87
Framingham score	**0.02**	**<0.0001**	**0.008**	**0.003**	**0.005**
Fib4	0.11	**0.008**	0.17	0.10	**0.03**
VACS score	0.38	**<0.0001**	**<0.0001**	**<0.0001**	**0.0001**

Abbreviations: ΔCD4; change in CD4 over time. P<0.05 highlighted in bold.

**Table 3:** Logistic regression of biomarkers (log transformed) with binary variables.

Biomarker	Odds Ratio	p-value
**CRP**		
AIDS	1.25 (1.01–1.55)	0.037
History of smoking	1.34 (1.08–1.66)	0.008
Currently smoking	1.36 (1.08–1.71)	0.008
**Cystatin**		
HCV	4.99 (1.5–16.6)	0.009
CD4<200	3.86 (1.07–13.9)	0.039
Currently smoking	3.58 (1.3–9.86)	0.014
BP meds	3.49 (1.26–9.65)	0.016
**D-dimer**		
HCV	1.63 (1.15–2.3)	0.005
CD4<200	1.83 (1.22–2.73)	0.003
Diabetes	1.83 (1.11–3.02)	0.017
CV disease	1.76 (1.09–2.85)	0.021
**IL-6**		
AIDS	1.45 (1.05–2.02)	0.025
CD4<200	2.95 (1.71–5.1)	<0.0001
History of smoking	1.89 (1.33–2.68)	<0.0001
Currently smoking	1.82 (1.28–2.58)	0.001
Diabetes	1.91 (1.05–3.45)	0.033
BP meds	1.53 (1.08–2.16)	0.016
HCV	1.7 (1.09–2.64)	0.019
**TNF-a**		
AIDS	2.3 (1.37–3.86)	0.002
CD4<200	2.62 (1.45–4.72)	0.001
History of smoking	1.78 (1.12–2.86)	0.017
Currently smoking	2.08 (1.29–3.37)	0.003

Abbreviations: CI, confidence interval. Only results which reached statistical significance (CI crossing 1) shown.

**Fig 1:**
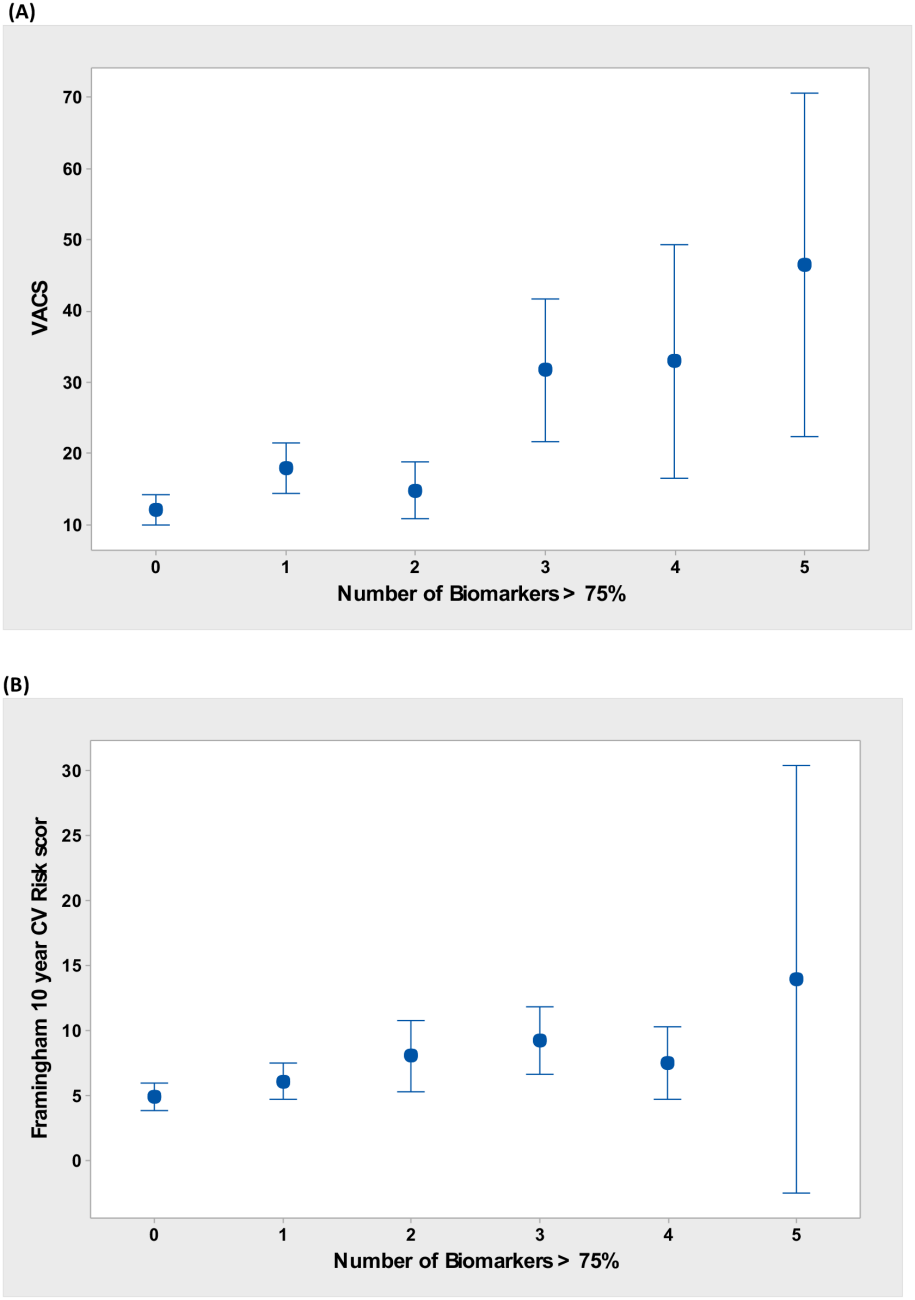
Predicting (A) VACS and (B) Framingham risk scores by number of biomarkers >75^th^ percentile(. Abbreviations: VACS, Veterans Aging Cohort Study score; CV, cardiovascular.

Increasing numbers of elevated biomarkers were associated with higher VACS scores (p = <0.0001, R-sq(adj) = 21.6%), particularly 3 or more [Fig 1]. Higher numbers of elevated biomarkers were also associated with higher Framingham risk scores, though the association was less robust than for the VACS score (p = 0.001, R-sq(adj) = 7.1%) [Fig 1].

## Discussion

A significant percentage of patients in this study had >1 serum biomarker level above the 75^th^ percentile, despite the fact that the majority were on antiretroviral therapy with completely suppressed viral loads and high CD4 counts. This is consistent with previous studies that have found that while some biomarker levels decrease after initiation of HAART, others remain elevated or even increase [13, 21, 22].

Several patient characteristics were more strongly associated with elevated biomarker levels than others. Increased age was associated with four of the five biomarkers studied, supporting the concept of ‘inflamm-aging’ or chronic, low grade increased inflammatory responses seen with advancing age that may be accentuated in patients with HIV [23]. Cigarette smoking, either past or current, was also seen to be consistently associated with elevated biomarkers, especially Il-6 and TNF-a. A prior study of biomarkers in HIV positive and HIV negative veterans in the Veterans Aging Cohort Study found that smoking was associated with a higher prevalence of elevated IL-6 (OR 1.67, 95% CI 1.17–2.37) but not D-dimer or sCD14 [24]. Long term follow up of the SMART and ESPRIT study cohorts, two large international cohorts with almost 10,000 participants, identified smoking and elevations in IL-6 and D-dimer as significant predictors of death, AIDS and significant non-AIDS events [25]. Given the high prevalence of smoking in the HIV-infected population (39–59%, versus about 19% in the general US population[26]), use of biomarkers for additional risk stratification may be helpful and may provide additional motivation for patients to participate in tobacco cessation programs.

Current CD4 counts of <200 cells/mm^3^ were associated with elevations in four out of five biomarker levels, and a diagnosis of AIDS was similarly associated with elevations of three out of the five biomarkers, consistent with previous studies showing an association between AIDS and inflammatory biomarkers [13,14]. Previously published data from the D.A.D and CASCADE studies showed a clear increase in the rate of death from non-AIDS causes in patients with CD4 counts of 200–349 mm3 compared with those with CD4 counts of >500 mm [3, 27]. In the SMART study, patients in the drug conservation group, on average, had a CD4 count that was 206 cells/mm^3^ lower than in the viral suppression group and an increased hazard ratio of 1.8 for death from any cause and 1.7 for major non-AIDS related disease [28]. TNF and IL-6 levels were strongly related to all-cause mortality in SMART and were strongly associated with CD4 counts of <200 cells/mm^3^ in our study. Finally, the recent landmark START study showed a clear benefit to immediate antiretroviral therapy in early asymptomatic HIV infection before a decline in CD4 counts with reductions in both AIDS-related and non-AIDS-related events [29]. Given the accumulating evidence that lower CD4 counts are associated with poor outcomes, it is plausible that low nadir CD4 counts and suboptimal CD4 gains on therapy may contribute to the systemic effects of inflammation via chronic activation and dysfunction of the innate immune system, and thus to increased long term morbidity and mortality.

We found limited biomarker correlation with typical cardiovascular risk factors such as diabetes or hypertension, though triglyceride levels were found to correlate with all five biomarkers measured [Table 2]. We had hypothesized that waist measurement would correlate with elevations in serum biomarkers given the known association between obesity, excess abdominal fat and cardio-metabolic risk, but no such correlation was found. We also found no correlation between use of aspirin or statins with biomarker levels, despite interest in the use of adjunctive anti-inflammatory drugs such as statins to modify outcomes in HIV patients [30], although our study was likely underpowered to find such an association.

Both the 10 year Framingham cardiovascular score and the VACS score were significantly associated with elevated biomarkers, particularly the VACS score. There was significant discordance between the estimation of cardiovascular risk using Framingham scores versus elevation of the hsCRP; only 3.2% of patients were found to have a Framingham risk score of >20, indicating a >20% risk of cardiovascular disease over 10 years, while almost 33% of patients had a hsCRP level of >3 indicating the same cardiovascular risk. Other studies of the Framingham risk estimation in populations thought to have higher levels of systemic inflammation, such as patients with chronic kidney disease and renal transplant patients, have found similar under-estimations of cardiovascular risk, with improvement in predictive power with the addition of inflammatory biomarkers [31,32]. It remains unclear whether cardiovascular risk estimation using the Framingham score or a biomarker such as hsCRP is accurate in the HIV infected population.

Almost one in five patients was found to have three or more biomarker levels above the 75^th^ percentile. As shown in Fig 1, as the number of elevated biomarkers above the 75^th^ percentile increased, there was a significant trend towards higher VACS scores (p = <0.0001, R-sq (adj) = 21.6%). A similar trend was also seen with the Framingham risk score although the model accounted for only 7% of the variability seen. Previous studies have assessed individual biomarkers of inflammation and coagulation, but the effects of these markers in aggregate is not known. A recent study of inflammatory biomarkers in HIV/HCV co-infected patients found that detectable HIV and HCV RNA was associated with a greater inflammatory burden score, defined as the presence of zero, one, two, or three or more elevated biomarkers of a panel of seven [33]. Our findings support their hypothesis that a composite measure of inflammation may be more appropriate for the HIV-infected population, given that the inflammatory response is suspected to represent overlapping contributions from immune dysregulation, coagulopathy and chronic viral replication.

There are several limitations to our study. Only 63% of the clinic patients were enrolled, raising the possibility of selection bias. Our clinic population is overwhelmingly male and white, which limits the generalizability of our findings to women and minority populations. The cross sectional nature of the study limited analysis of outcomes, although the Framingham and VACS risk scores were meant as surrogate measures of outcomes. Each biomarker was assayed only once raising concerns for variability (inherent in any laboratory assay) and day to day variability of biomarkers in each patient.

## Conclusion

In this clinic population of HIV-infected patients with high rates of viral suppression and immunological stability, elevations in inflammatory biomarkers are present in a significant percentage of patients. Age, cigarette smoking, and CD4 counts of <200 cells/mm^3^ were associated with elevated serum biomarkers. Those with a higher aggregate number of elevated biomarkers had higher VACS scores and Framingham scores, suggesting that a higher inflammatory burden may contribute to long term morbidity and mortality in this population. Biomarker measurement in combination with standard risk assessment scores may ultimately be a valuable screening tool to further assess patient risks for morbidity and mortality, especially those with good viral and immunological control.

## References

[1] Effros RB, Fletcher CV, Gebo K, Halter JB, Hazzard WR, Home FM, et al Aging and infectious diseases: workshop on HIV infection and aging: what is known and future research directions. Clin Infect Dis. 2008 8 15;47(4):542–53. 10.1086/590150 18627268 PMC3130308

[2] Samji H, Cescon A, Hogg RS, Modur SP, Althoff KN, Buchacz K, et al Closing the Gap: Increases in life expectancy among treated HIV-positive individuals in the United States and Canada. PLoS One. 2013 12 18;8(12):e81355 10.1371/journal.pone.0081355 24367482 PMC3867319

[3] Currier JS, Taylor A, Boyd F, Dezii CM, Kawabata H, Burtcel B, et al Coronary heart disease in HIV-infected individuals. J Acquir Immune Defic Syndr. 2003 8 1;33(4):506–512. 12869840 10.1097/00126334-200308010-00012

[4] Salmon-Ceron D, Rosenthal E, Lewden C, Bouteloup V, May T, Burty C, et al Emerging role of hepatocellular carcinoma among liver-related causes of deaths in HIV-infetced patients: The French national Mortalité 2005 study. J Hepatol. 2009 4;50(4):736–45. 10.1016/j.jhep.2008.11.018 19231018

[5] Triant VA, Brown TT, Lee H, Grinspoon SK. Fracture prevalence among human immunodeficiency virus (HIV)-infected individuals versus non-HIV-infected patients in a large U.S. healthcare system. J Clin Endocrinol Metab. 2008 9;93(9):3499–504. 10.1210/jc.2008-0828 18593764 PMC2567857

[6] Shiels MS, Pfeiffer RM, Gail MH, Hall HI, Li J, Chaturvedi AK, et al Cancer burden in the HIV-infected population in the United States. J Natl Cancer Inst. 2011 5 4;103(9):753–62. 10.1093/jnci/djr076 21483021 PMC3086877

[7] Desquilbet L, Jacobson LP, Fried LP, Phair JP, Jamieson BD, Holloway M, et al HIV-1 infection is associated with an earlier occurrence of a phenotype related to frailty. J Gerontol A Biol Sci Med Sci. 2007 11;62(11):1279–86. 18000149 10.1093/gerona/62.11.1279

[8] Guaraldi G, Orlando G, Zona S, Menozzi M, Carli F, Garlassi E, et al Premature age-related comorbidities among HIV-infected persons compared with the general population. Clin Infect Dis. 2011 12;53(11):1120–6. 10.1093/cid/cir627 21998278

[9] Deeks SG, Tracy R, Douek DC. Systemic effects of inflammation on health during chronic HIV infection. Immunity. 2013 10 17;39(4):633–45. 10.1016/j.immuni.2013.10.001 24138880 PMC4012895

[10] Kuller LH, Tracy R, Belloso W, De Wit S, Drummond F, Lane HC, et al Inflammatory and coagulation biomarkers and mortality in patients with HIV infection. PLoS Med. 2008 10 21;5(10):e203 10.1371/journal.pmed.0050203 18942885 PMC2570418

[11] Nixon DE, Landay AL. Biomarkers of immune dysfunction in HIV. Curr Opin HIV AIDS. 2010 11;5(6):498–503. 10.1097/COH.0b013e32833ed6f4 20978393 PMC3032605

[12] Neuhaus J, Jacobs DR Jr, Baker JV, Calmy A, Duprez D, La Rosa A, et al Markers of inflammation, coagulation and renal function are elevated in adults with HIV infection. J Infect Dis. 2010 6 15;201(12):1788–95. 10.1086/652749 20446848 PMC2872049

[13] Boulware DR, Hullsiek KH, Puronen CE, Rupert A, Baker JV, French MA, et al Higher levels of CRP, D-dimer, IL-6 and hyaluronic acid before initiation of antiretroviral therapy (ART) are associated with increased risk of AIDS or death. J Infect Dis. 2011 6 1;203(11):1637–46. 10.1093/infdis/jir134 21592994 PMC3096784

[14] McComsey GA, Kitch D, Sax PE, Tierney C, Jahed NC, Melbourne K, et al Associations of inflammatory markers with AIDS and non-AIDS Clinical events after Initiation of antiretroviral therapy: AIDS Clinical Trials Group A5224s, a substudy of ACTG A5202. J Acquir Immune Defic Syndr. 2014 2 1;65(2):167–74. 10.1097/01.qai.0000437171.00504.41 24121755 PMC3943548

[15] Justice AC, Freiberg MS, Tracy R, Kuller L, Tate JP, Goetz MB, et al Does an index composed of clinical data reflect effects of inflammation, coagulation, and monocyte activation on mortality among those aging with HIV? Clin Infect Dis. 2012 4;54(7):984–94. 10.1093/cid/cir989 22337823 PMC3297653

[16] Guzmán-Fulgencio M, Medrano J, Rallón N, Echeverria-Urabayen A, Miguel Benito J, Restrepo C, et al Soluble markers of inflammation are associated with Framingham scores in HIV-infected patients on suppressive antiretroviral therapy. J Infect. 2011 11;63(5):382–90. 10.1016/j.jinf.2011.08.006 21855573

[17] Justice AC, McGinnis KA, Skanderson M, Chang CC, Gibert CL, Goetz MB, et al Towards a combined prognostic index for survival in HIV infection: the role of 'non-HIV' biomarkers. HIV Med. 2010 2;11(2):143–51. 10.1111/j.1468-1293.2009.00757.x 19751364 PMC3077949

[18] Justice AC, Modur SP, Tate JP, Althoff KN, Jacobson LP, Gebo KA, et al Predictive accuracy of the Veterans Aging Cohort Study index for mortality with HIV infection: a North American cross cohort analysis. J Acquir Immune Defic Syndr. 2013 2 1;62(2):149–63. 10.1097/QAI.0b013e31827df36c 23187941 PMC3619393

[19] D’Agostino RB Sr. Cardiovascular risk estimation in 2012: lessons learned and applicability to the HIV population. J Infect Dis. 2012 6;205 Suppl 3:S362–7. 10.1093/infdis/jis196 22577209 PMC3349294

[20] Framingham Risk Assessment Tool, National Heart Lung and Blood Institute. Available: http://www.cvdrisk.nhlbi.nih.gov.

[21] Baker JV, Neuhaus J, Duprez D, Kuller LH, Tracy R, Belloso WH, et al Changes in inflammatory and coagulation biomarkers: a randomized comparison of immediate versus deferred antiretroviral therapy in patients with HIV infection. J Acquir Immune Defic Syndr. 2011 1 1;56(1):36–43. 10.1097/QAI.0b013e3181f7f61a 20930640 PMC3005856

[22] Palella FJ Jr, Gange SJ, Benning L, Jacobson L, Kaplan RC, Landay AL, et al Inflammatory biomarkers and abacavir use in the Women's Interagency HIV Study and the Multicenter AIDS Cohort Study. AIDS. 2010 7 17;24(11):1657–65. 10.1097/QAD.0b013e3283389dfa 20588104 PMC3514460

[23] Deeks SG. HIV infection, inflammation, immunosenescence, and aging. Annu Rev Med. 2011;62:141–55. 10.1146/annurev-med-042909-093756 21090961 PMC3759035

[24] Armah KA, McGinnis K, Baker J, Gibert C, Butt AA, Bryant KJ, et al HIV status, burden of comorbid disease, and biomarkers of inflammation, altered coagulation, and monocyte activation. Clin Infect Dis. 2012 7;55(1):126–36. 10.1093/cid/cis406 22534147 PMC3493182

[25] Miller CJ, Baker JV, Bormann AM, Erlandson KM, Huppler Hullsiek K, Justice AC, et al Adjudicated morbidity and mortality outcomes by age among individuals with HIV infection on suppressive antiretroviral therapy. PLoS One. 2014 4 11;9(4):e95061 10.1371/journal.pone.0095061 24728071 PMC3984283

[26] Shirley DK, Kaner RJ, Glesby MJ. Effects of smoking on non-AIDS-related morbidity in HIV-infected patients. Clin Infect Dis. 2013 7;57(2):275–82. 10.1093/cid/cit207 23572487 PMC3689343

[27] Phillips AN, Neaton J, Lundgren JD. The role of HIV in serious diseases other than AIDS. AIDS. 2008 11 30;22(18):2409–18. 10.1097/QAD.0b013e3283174636 19005264 PMC2679976

[28] Strategies for Management of Antiretroviral Therapy (SMART) Study Group, El-Sadr WM, Lundgren J, Neaton JD, Gordin F, Abrams D, et al CD4+ count-guided interruption of antiretroviral therapy. N Engl J Med. 2006 11 30;355(22):2283–96. 17135583 10.1056/NEJMoa062360

[29] INSIGHT START study group, Lundgren JD, Babiker AG, Gordin F, Emery S, Grund B, et al Initiation of antiretroviral therapy in early asymptomatic HIV infection. N Engl J Med. 2015 8 27;373(9):795–807. 10.1056/NEJMoa1506816 26192873 PMC4569751

[30] Eckard AR, Jiang Y, Debanne SM, Funderburg NT, McComsey GA. Effect of 24 weeks of statin therapy on systemic and vascular inflammation in HIV-infected subjects receiving antiretroviral therapy. J Infect Dis. 2014 4 15;209(8):1156–64. 10.1093/infdis/jiu012 24415784 PMC3969551

[31] Silver SA, Huang M, Nash MM, Prasad GV. Framingham risk score and novel cardiovascular risk factors underpredict major cardiac events in kidney transplant recipients. Transplantation. 2011 7 27;92(2):183–9. 10.1097/TP.0b013e31821f303f 21558986

[32] Chen SC, Su HM, Tsai YC, Huang JC, Chang JM, Hwang SJ, et al Framingham risk score with cardiovascular events in chronic kidney disease. PLoS One. 2013;8(3):e60008 10.1371/journal.pone.0060008 23527293 PMC3603980

[33] Armah KA, Quinn EK, Cheng DM, Tracy RP, Baker JV, Samet JH, et al Human immunodeficiency virus, hepatitis C, and inflammatory biomarkers in individuals with alcohol problems: a cross-sectional study. BMC Infect Dis. 2013 8 29;13:399 10.1186/1471-2334-13-399 23987993 PMC3848623

